# Presence of cutaneous lesion is a poor prognostic factor in patients with smoldering-type adult T-cell leukemia-lymphoma

**DOI:** 10.1186/1742-4690-8-S1-A35

**Published:** 2011-06-06

**Authors:** Kentaro Yonekura, Atae Utsunomiya, Kazuhiro Kawai, Yoshifusa Takatsuka, Shogo Takeuchi, Masahito Tokunaga, Ayumu Kubota, Tamotsu Kanzaki, Youhei Uchida, Takuro Kanekura

**Affiliations:** 1 Department of Dermatology, Kagoshima University Graduate School of Medical and Dental Sciences, Kagoshima, Kagoshima, 890-8520, Japan; 2 Department of Dermatology, Imamura Bun-in Hospital, Kagoshima, Kagoshima, 890-0064, Japan; 3 Department of Hematology, Imamura Bun-in Hospital, Kagoshima, Kagoshima, 890-0064, Japan

## Background

Prognosis of indolent types of adult T-cell leukemia-lymphoma (ATL) including the smoldering type was recently reported to be poorer than that shown in previous studies. Prognostic factors of smoldering-type ATL have not been defined. Cutaneous-type, which is defined as cases of smoldering type predominantly involve skin with or without peripheral blood involvement has been proposed as a distinct clinical subtype. Prognosis of cutaneous-type ATL was reported to be poorer than that of smoldering-type without cutaneous involvement.

## Aim

To determine prognostic factors for survival and disease progression of smoldering-type ATL.

## Patients

Thirty-one patients with smoldering-type ATL including 21 with cutaneous lesion.

## Methods

Multivariate Cox proportional hazards model was used to identify variables associated with survival and disease progression. Peripheral blood abnormal lymphocytes (<5% vs ≥5%), serum lactate dehydrogenase (normal vs high), albumin level (<4 vs ≥4 g/dl), and cutaneous lesion (none vs present) were used as variables. Overall survival (OS) and progression-free survival (PFS) were estimated using the Kaplan-Meier method and compared using the log-rank test.

## Results

In the multivariate analysis, presence of cutaneous lesion was the significant prognostic factor for PFS (hazard ratio, 8.69; 95% confidence interval, 1.4-54.0; P = 0.02). Peripheral blood abnormal lymphocytes, serum lactate dehydrogenase, or albumin level was not significant. None of the variables was significantly associated with OS in the multivariate analysis. OS (P = 0.117) and PFS (P = 0.089) of the patients with cutaneous lesion were worse as compared to those of the patients without cutaneous lesion, though statistically not significant.

## Conclusions

In this study we confirmed that presence of cutaneous lesion is an independent prognostic factor in patients with smoldering-type ATL. Cases of smoldering-type with cutaneous lesion should be classified as cutaneous-type ATL irrespective of the peripheral blood involvement.

